# Inducing and Recording Acute Stress Responses on a Large Scale With the Digital Stress Test (DST): Development and Evaluation Study

**DOI:** 10.2196/32280

**Published:** 2022-07-15

**Authors:** Matthias Norden, Amin Gerard Hofmann, Martin Meier, Felix Balzer, Oliver T Wolf, Erwin Böttinger, Hanna Drimalla

**Affiliations:** 1 Faculty of Technology Bielefeld University Bielefeld Germany; 2 Digital Health Center Hasso Plattner Institute University of Potsdam Potsdam Germany; 3 Institute of Medical Informatics Charité – Universitätsmedizin Berlin Corporate member of Freie Universität Berlin and Humboldt-Universität zu Berlin Berlin Germany; 4 Department of Cognitive Psychology Institute of Cognitive Neuroscience, Faculty of Psychology Ruhr University Bochum Bochum Germany; 5 Hasso Plattner Institute for Digital Health at Mount Sinai Icahn School of Medicine at Mount Sinai New York City, NY United States

**Keywords:** stress induction, smartphone, stress reactivity, Trier Social Stress Test, TSST, remote, video recording, acute stress, digital health, mobile health, mHealth, mobile phone

## Abstract

**Background:**

Valuable insights into the pathophysiology and consequences of acute psychosocial stress have been gained using standardized stress induction experiments. However, most protocols are limited to laboratory settings, are labor-intensive, and cannot be scaled to larger cohorts or transferred to daily life scenarios.

**Objective:**

We aimed to provide a scalable digital tool that enables the standardized induction and recording of acute stress responses in outside-the-laboratory settings without any experimenter contact.

**Methods:**

On the basis of well-described stress protocols, we developed the Digital Stress Test (DST) and evaluated its feasibility and stress induction potential in a large web-based study. A total of 284 participants completed either the DST (n=103; 52/103, 50.5% women; mean age 31.34, SD 9.48 years) or an adapted control version (n=181; 96/181, 53% women; mean age 31.51, SD 11.18 years) with their smartphones via a web application. We compared their affective responses using the international Positive and Negative Affect Schedule Short Form before and after stress induction. In addition, we assessed the participants’ stress-related feelings indicated in visual analogue scales before, during, and after the procedure, and further analyzed the implemented stress-inducing elements. Finally, we compared the DST participants’ stress reactivity with the results obtained in a classic stress test paradigm using data previously collected in 4 independent Trier Social Stress Test studies including 122 participants overall.

**Results:**

Participants in the DST manifested significantly higher perceived stress indexes than the Control-DST participants at all measurements after the baseline (*P*<.001). Furthermore, the effect size of the increase in DST participants’ negative affect (*d*=0.427) lay within the range of effect sizes for the increase in negative affect in the previously conducted Trier Social Stress Test experiments (0.281-1.015).

**Conclusions:**

We present evidence that a digital stress paradigm administered by smartphone can be used for standardized stress induction and multimodal data collection on a large scale. Further development of the DST prototype and a subsequent validation study including physiological markers are outlined.

## Introduction

### Relevance and Rationale

Psychosocial stress is a major risk factor for the development of physical and mental illnesses, including hypertension, depression, and anxiety [1]. Valuable insights into its causes and consequences have been gained through experimental stress paradigms during which acute stressors are used to induce a psychosocial stress reaction. For example, such stress induction paradigms have been successfully used to investigate the effects of acute stress on the brain [2], hormonal and inflammatory reactivity [3], memory [4], and social cognition and behavior [5].

Applying controlled stress induction paradigms also enables the investigation of prevention and intervention strategies. For example, in a recent study, Het et al [6] used a classic stress paradigm to study the effects of an inpatient treatment on acute stress reactivity in women with eating disorders. In addition, controlled stress induction procedures play an important role in the development of objective stress detection methods [7,8] as they strongly rely on highly qualitative and representative data sets obtained through stress induction experiments [9].

### Current Stress Paradigms and Their Limitations

Currently, most stress induction paradigms are limited in their scalability (ie, applicable across a large number of participants and distances) and, thus, cannot be easily used to gather large volumes of stress-related data. Furthermore, many of these have not been replicated outside the laboratory to verify the laboratory findings in outside-the-laboratory settings [10]. To overcome these limitations, a new standardized and validated stress induction paradigm is needed.

The Trier Social Stress Test (TSST) [11] is considered the gold standard in human experimental stress research, having been applied >4000 times including different populations and age groups [12]. Participants have to complete a 5-minute mock job interview and a 5-minute mental arithmetic task in front of an evaluating committee. This procedure requires a laboratory setup, an experimenter, and 2 actors playing the committee, making the TSST costly and unfeasible for large-scale application. In addition, the impact of the different methodological elements (eg, panel composition) on the stress reaction and the relatively small sample sizes complicate the reproducibility of the findings [13-15]. Furthermore, the experimental setting might lead to stress responses that differ from acute stress experienced in daily life.

Several adaptations have been made to provide less costly and laborious versions, but they still require human resources (eg, TSST for groups) or additional equipment (eg, virtual reality TSST or e-TSST) and have not been tested in nonlaboratory settings. Recently, 2 studies applied a web-based version of the TSST during which adolescent [16] or adult [17] participants joined judges and experimenters on a web-based videoconferencing platform without any in-person assessment. The responses to these web-delivered versions were consistent with standard in-person responses although the paradigm was conducted remotely. This highlights the possibility of assessing stress reactivity outside a research laboratory. However, the entire procedure still depends on live interactions between the committee, the participant, and the experimenter.

Stressors that enable the investigation of stress responses without direct experimenter contact have been developed for imaging scenarios [18]. Using their Imaging Paradigm for Evaluative Social Stress, Fehlner et al [19] showed that delivering short spoken answers to selected topics in front of a prerecorded audience and additional framings induced robust stress responses. This indicates that psychosocial stress can also be induced by making the participants believe they are exposed to some kind of social evaluation without direct experimenter interaction.

The Montreal Imaging Stress Task (MIST) [20] supports this assumption. It comprises computerized mental arithmetic tasks with an induced failure component and social pressure elements. However, these paradigms have only been tested within imaging laboratory settings where experimenters were still present and performed potentially stressful measurements. Thus, the stress induction might be influenced by the imaging setting and the experimenter’s role during the procedure.

Many other well-described stress paradigms (eg, the CO2 challenge test and the socially evaluated cold pressor test [21]) are dependent on laboratory settings, build on physical stressors, and require human resources or additional equipment [22]. Other paradigms (eg, the Paced Auditory Serial Addition Task [23] and Stroop test [24]) would theoretically be applicable outside the laboratory but lack the possibility to collect multimodal behavior data (eg, facial expressions and voice recordings) of the stress response. To the best of our knowledge, there is currently no standardized and validated digital stress paradigm that can be carried out without an experimenter and collect multimodal video data of participants in stressed conditions. Therefore, we conceptualized and developed a completely digital stress test to address the need for an innovative, standardized, and validated stress induction protocol.

### Digital Stress Test

The Digital Stress Test (DST) is primarily intended as a digital research tool. Importantly, we did not aim to develop a direct stress measurement or therapeutic tool. Instead, the DST enables researchers to gain additional insights into acute stress responses by making stress studies scalable and transferable to outside-the-laboratory settings and collecting stress-relevant video data at the same time. Thus, the DST is designed as an easy-to-use smartphone web application where participants conduct the study (via the internet) without any direct communication with researchers or additional resources required (ie, wearables or native app downloads).

It combines different well-known stress induction principles of classic stress paradigms and adapts them to a digital setting. According to a meta-analysis of psychological stress paradigms by Dickerson and Kemeny [25], a robust and reliable stress response can be induced by acute or chronic threats to social status, particularly when conditions are uncontrollable. Most likely, this would occur when failure or poor performance could reveal a lack of ability. Both principles have been proven effective in state-of-the-art stress paradigms and will be used as the basis for the digital stress induction paradigm.

Second, the DST aims to collect multimodal behavior data (ie, facial and voice cues) that can be used to build a basic data set for further (machine learning) analysis. Therefore, the embedded stress induction procedure will include a naturalistic speaking part (ie, comparable with daily speaking).

### Objectives and Hypotheses

The aim of this study was to develop the first prototype of a DST web application and assess its feasibility as well as its stress induction potential. Therefore, we also provided a neutral version called the Control-DST (C-DST) that can be used similarly in web-based settings. We hypothesized that the DST would elicit a stronger stress response compared with the neutral condition. In addition, we placed our results in the context of previous studies conducting the gold-standard paradigm (TSST).

This paper is organized as follows: in the *Methods* section, we describe the concept and development of the DST and its control version. Furthermore, we provide details of the large-scale web-based study conducted to evaluate the feasibility and stress induction potential of the DST. In the *Results* section, we present statistical evidence for the stress induction potential of the DST. Finally, in the *Discussion* section, we discuss our results and potential limitations in light of previous work and outline plans for future research.

## Methods

### Concept and Development of the DST

We first describe the underlying stress induction paradigm as well as its adaptation for the development of a suitable control condition. We include illustrations of the first DST and C-DST prototypes and outline the technological aspects. Before starting the web-based evaluation study, we conducted a pilot study to finalize the prototypes based on participants’ feedback.

#### Concept of the DST

##### Overview

**Figure 1:**
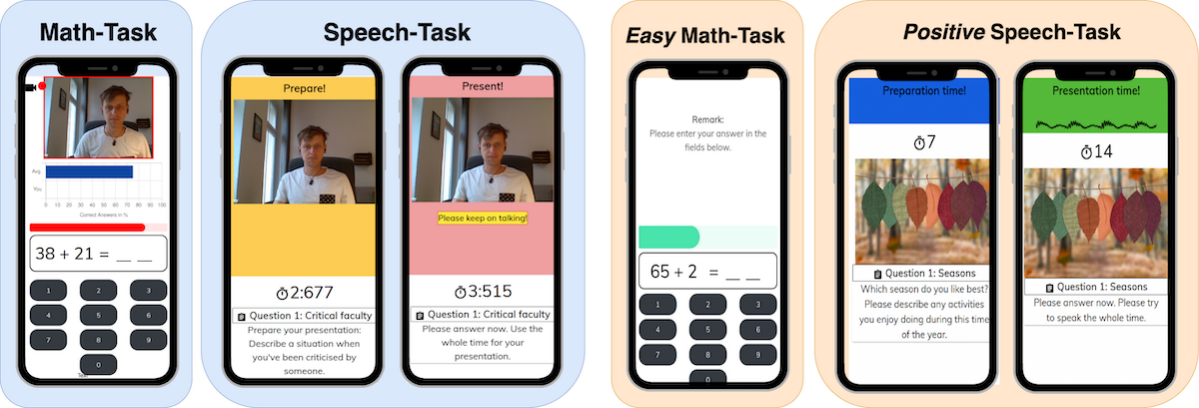
Screenshots of the Digital Stress Test (blue boxes) and Control - Digital Stress Test (orange boxes) tasks. The left box illustrates the Math-Task in each version, and the right box shows the preparation and presentation parts of the respective Speech-Task.

The paradigm consists of an arithmetic calculation and a free speech part and is framed as a cognitive-verbal performance test. Screenshots of the DST and its control condition are shown in Figure 1. The complete web application procedures can be seen in Multimedia Appendices 1 and 2. Presentation versions of the most recent DST and C-DST without any data saving can be found at their respective websites [26,27].

To elicit a robust acute psychosocial stress reaction, the DST procedure comprises multiple elements of social-evaluative threat and uncontrollability [11,25].

##### Framing

The DST is introduced as a research tool for “behavior analysis while performing a cognitive-verbal performance test,” indicating that the individual performance of the participant is tested. To further increase the social-evaluative threat, they are informed that they will be recorded through the front camera of their smartphones and that these recordings are being analyzed to assess their individual resilience.

The participants record a short test video that claims to calibrate the implemented algorithm and shall increase the credibility of the automated behavior analysis.

The cognitive task is framed as a simple calculation task that a fake comparison group (based on age and gender) apparently solved with an average of 75% correct answers. This intends to emphasize the expected results and introduce the participants to the permanent social comparison in the upcoming calculation task, as done in the MIST [20].

##### Arithmetic Calculation Task (*Math-Task*)

The task comprises elements of the MIST [20] protocol adapted to the smartphone setting and enhanced with several other stress-inducing elements. After a countdown, the participants are required to solve simple calculation tasks consisting of addition, subtraction, multiplication, and division of 2 numbers ranging from 1 to 99 with solutions ranging from 1 to 99. The participants need to type their solution on a number field within the given time limit. If the response is wrong or no response is recorded within the time limit, negative feedback is presented (“Wrong answer!” or “Too slow!”) and the background color changes to red. After a correct response, the next calculation task is presented immediately. The time limit for each calculation is marked using a red expiring progress bar.

A continuous failure rate is being provoked. For the first task, the time limit is set to 3 seconds. If the participant answers a series of 3 consecutive arithmetic tasks correctly, the time limit is shortened by 10%. In addition, for the following 4 tasks, the numbers of the input field are swapped randomly to increase the difficulty and uncontrollability. If the participant answers a series of 3 consecutive tasks incorrectly (or not at all), the time limit is extended by 10%. If the participant does not give any input for 5 consecutive tasks, feedback indicating the relevance of the study is displayed, and the next task is chosen to be easily solvable (ie, a summation task). This intends to ensure ongoing participation.

During the Math-Task, the percentage of correct responses is continuously displayed and compared with the fixed average of the participants’ age- and gender-related groups in a bar chart. As the achieved percentage of correct answers in the comparison group was claimed to be 75%, which usually exceeds the current percentage of the participant because of the implemented difficulty, this continually reminds the participant of failing.

In addition, the front camera of the smartphone is activated, and the recorded video is displayed directly on the upper half of the screen during the entire Math-Task. This intends to remind the participants that they are being recorded and apparently analyzed while failing in a school-like performance task.

The participants do not know how long the Math-Task takes to increase a feeling of uncontrollability. After 1.5 minutes, the Math-Task automatically stops. The participants see their final percentage of correct answers compared with the fabricated age- and gender-related average and are reminded that “only serious results can be used for this study,” emphasizing the relevance of the participants’ performance.

##### Free Speech Task (*Speech-Task*)

The second part is the Speech-Task, which further extends the social-evaluative threat through a presentation-like situation and enables the recording of stress-relevant voice cues. The participants are reminded that their verbal skills will be assessed. They are instructed to prepare structured and convincing verbal answers to standard job interview questions. They are not told how many questions will follow, making the length of this task unpredictable.

The Speech-Task includes 3 inconvenient answering scenarios (eg, “Describe a situation when you’ve been criticized by someone!”) that are based on a previous study by Fehlner et al [19]. For each scenario, they are given 10 seconds to prepare and 20 seconds to present their speech. The participants are reminded to use the entire time for their presentation.

A countdown indicates the time for preparation and presentation, intending to pressure the participants. During their presentations, the background color of the entire screen blinks red to visually distract and agitate the participants.

The smartphone’s front camera is activated, and the recorded video is displayed on the upper half of the screen during the preparation and presentation periods. In addition, a voice visualization is included in the presentation parts. After 1 second without recorded noise input signal, the participant is reminded to keep on talking, increasing the credibility of the behavior analysis and pressuring the participants, as done by the experimenters in the TSST paradigm [11,13]**.**

#### Concept of the C-DST

##### Overview

We also developed a control version of the DST that resembles its structure and procedure but differs in terms of the stress induction elements (Figure 1, right side). We changed the tasks and framings to be less stressful, as done for the placebo TSST [28] and friendly TSST [29]. The provided information on the study’s background, privacy, and data protection aspects, as well as the performance task framing in the beginning, remains exactly the same to have a comparable baseline. The differences are outlined in the following sections.

##### *Friendly* Framing

The participants are informed that they are part of a control group and that they will not be video recorded. No recording of a test video or any additional framing of an automated behavior analysis takes place. The participants are not told that their individual performance results will be compared with those of other participants, and no fictive average result scores are displayed.

##### *Easy* Math-Task

The calculation tasks in the C-DST are generated in the same way as in the DST but only with summation tasks. The time limit for the first task is set to 5 seconds. The time adaptation algorithm is designed to enable more correct responses—as soon as the participant answers 1 task incorrectly (or not at all), the time limit is extended by 10%.

Only if the participant answers a series of 4 consecutive tasks correctly the time limit is shortened by 10%. In contrast to the DST, the numbers of the input field are not swapped for the following task.

The provided feedback is chosen to be encouraging (ie, the screen color changes to green for correct answers and does not change for wrong answers). The time limit is marked using a green progress bar. Neither a fake comparison with other participants’ results nor any live recording through the front camera is displayed.

##### *Positive* Speech-Task

As opposed to the DST Speech-Task, the *positive* Speech-Task is not introduced as an assessment of the participants’ verbal skills. The answering scenarios include only neutral topics (eg, “Which season do you like best? Please describe any activities you enjoy doing during this time of the year!”).

Instead of displaying the live-recorded video of the front camera on the upper half of the screen, a neutral image suiting the question is shown. No further distraction through a red blinking background takes place, and the colors are chosen to be calming.

#### Technological Aspects

**Figure 2:**
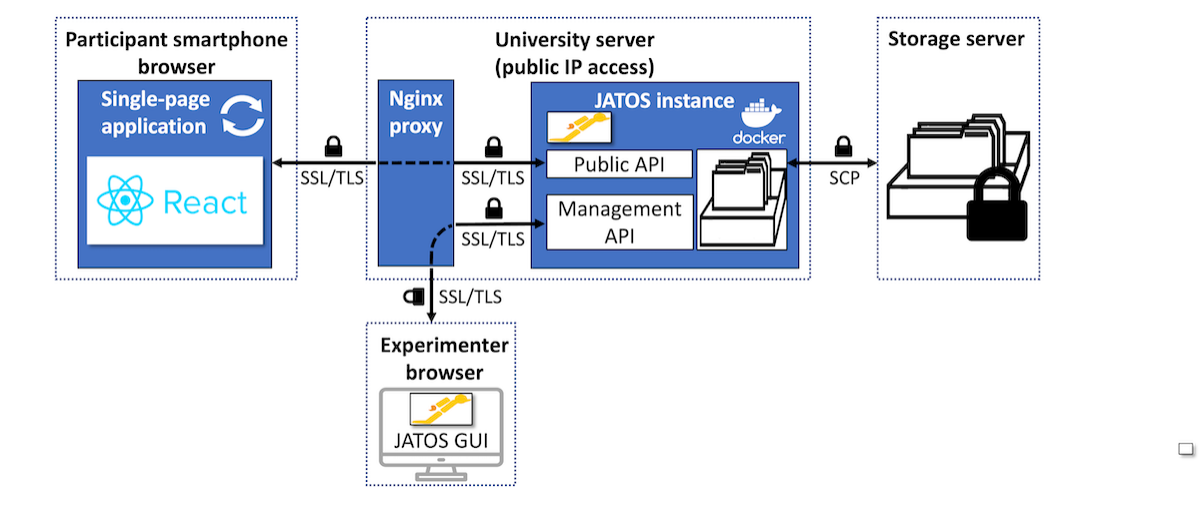
System architecture of the test applications. The Digital Stress Test and Control - Digital Stress Test work as single-page web applications within the participant's smartphone browser (left side). The single-page applications are hosted on an web-based reachable university server (center) using the open-source web study management system JATOS [28] within a Docker container. API: application programming interface; GUI: graphical user interface; SCP: secure copy protocol; SSL: secure socket layer; TLS: transport layer security.

The system architecture of the applications is shown in Figure 2. The DST and C-DST were developed as single-page web applications using the JavaScript framework React.js. The source code of the most recent versions is publicly accessible at the website [30]. The applications run on standard browsers and are hosted on a university server that allows for public IP access using the open-source study management system JATOS [31] within a Docker container. JATOS exposes a public application programming interface (API) that is called with a wrapper library and handles requests from a participant’s browser (eg, fetch and upload data). In addition, it provides a management API to handle requests from the experimenter’s browser via the JATOS graphical user interface. More detailed information on the JATOS architecture can be found in the study by Lange et al [31].

In this study, only fully anonymized data were collected. We disabled the recording of videos but only streamed them within the participant’s smartphone browser as the focus of this study was testing and validating the digital stress induction procedure. Owing to the capability and future plans to also collect sensitive and potentially identifiable video data, we implemented several security measures.

Nginx (Nginx, Inc) is used on the publicly reachable university server to ensure Secure Sockets Layer encryption, and it only responds to https requests for calls to both the public and management JATOS APIs. Participant data are only temporarily stored on the web server and directly transferred to a secure storage server via secure copy protocol after the test ends. All (remaining) data are deleted automatically from the web server in short time intervals. We have already received ethics approval for our data storage concept. For the future, we also plan to implement a client-side encryption of participant data files that takes place already within the web applications and can only be decrypted using private keys from the secure storage server.

#### Pilot Study

We conducted a pilot study with 49 participants performing either the DST (21/49, 43%) or the C-DST (28/49, 57%) web application. On the basis of their feedback, we adjusted major usability issues that were caused by different browsers and smartphones and fixed technological bugs. We aimed for a comprehensive study introduction and consenting and debriefing information and modified the wording accordingly.

### Evaluation of the DST

To assess the feasibility and stress induction potential of the DST, we first conducted a large web-based study. Participants in this web-based study performed either the DST or the C-DST and filled out several questionnaires regarding their affective responses. The effect sizes of the affective changes indicated by the DST participants in this web-based study were then compared with results obtained in previous studies performing the laboratory gold-standard paradigm (TSST).

#### Participants and Recruitment for the Web-Based Study

##### Overview

Participants were recruited via web-based publication of the study link in the university and study participation mailing lists, social networks (eg, Twitter and Facebook), podcasts, and websites. The study was conducted for 2 weeks, from February 10 to February 24, 2021. Within this period, 547 participants performed either the DST (300/547, 54.8%) or C-DST (247/547, 45.2%). For the evaluation of subjective stress parameters, we excluded participants with incomplete tests (229/547, 41.9%), previous self-reported knowledge of the framing (13/547, 2.4%), self-reported usability issues (19/547, 3.5%), or unrealistic procedure duration (2/547, 0.4%).

##### Web-Based Study Procedure

**Figure 3:**
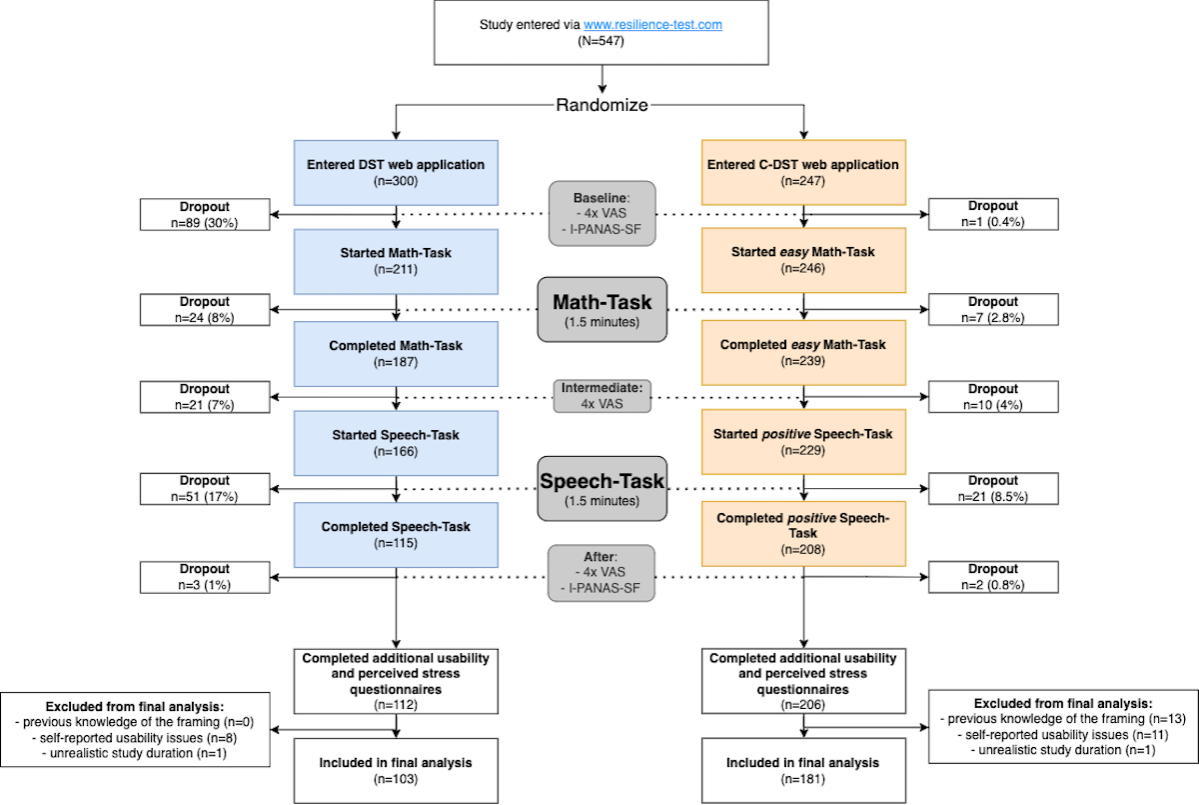
Web-based study design, drop-outs, and collected data. The participants are randomly assigned to either the Digital Stress Test (blue boxes) or Control - Digital Stress Test (orange boxes) web application using the provided study link. The participants answer the same questionnaires within the respective web application and additional questionnaires on an external website afterward. I-PANAS-SF: international Positive and Negative Affect Schedule Short Form; VAS: Visual Analogue Scale.

The design of the web-based study is shown in Figure 3. The entire procedure takes place on the screen of the participants’ smartphones and takes approximately 5 to 10 minutes. Using the provided study link, the participants were randomly forwarded to either the stress or control paradigm. We adjusted the randomization algorithm to prefer the DST when we analyzed the dropout rates after the first week. However, most participants (479/547, 87.6%) performed the study within the first week because of the publication in a widespread German politics podcast.

During the pretest part of the web application, the participants were introduced to the study background, upcoming procedure, and privacy and data protection aspects. Assessments of the perceived stress level took place before, between, and after the 2 tasks using built-in questionnaires.

After completing both tasks, the participants were debriefed and linked to additional usability and follow-up questionnaires on an external website [32]. The participants could quit the study at any time (eg, by closing the browser).

##### Web-Based Study Data Collection

General demographic information, including age and gender, was obtained in the first part of the session. In addition, previous participation was asked about to exclude duplicated results with previous knowledge and confounding factors.

To assess the perceived stress levels, the participants completed several self-report questionnaires (Textbox 1).

To further compare the feasibility of the 2 paradigms, several pieces of metadata were stored during the procedure. This included the performance during the Math-Task and the study progress (ie, how much time the participants spent on different parts of the application and at which part they cancelled).

The participants were linked to additional questionnaires at the end of the web application. These included several questions on usability aspects (eg, problems with the correct camera and problems with the Visual Analogue Scale [VAS]) as well as the opportunity to provide open feedback. Furthermore, we asked the participants to rate their perceived stress regarding specific parts of the applications on a VAS.

**In-app self-report questionnaires completed by the participants.**

**Self-report questionnaires**

- The international short form of the Positive and Negative Affect Schedule (PANAS) [33] was applied to assess positive and negative affect in the beginning (baseline) and after solving both tasks (posttest assessment). The PANAS is a well-validated and reliable tool to assess the participants’ mood that has been applied in various studies on mood changes [34]. The participants indicate the intensity of 10 feelings and emotions on a 5-point Likert scale. The items can be subdivided into negative affect (NA; consisting of 5 items) and positive affect (PA; consisting of 5 items). We used the mean scores for both affects and normalized them for the number of items (ie, PA and NA outcomes ranging from 1 to 5 for each time point).
- Visual Analogue Scales (VAS) regarding 4 different dimensions of stress (feeling *stressed, frustrated, overstrained*, and *ashamed*) were obtained in the beginning, between the 2 tasks, and in the end. The VAS is a common instrument to measure characteristics that cannot be easily measured directly and is often used for pain, stress, or other subjective experiences [35]. The participants indicate how much they are perceiving specific feelings at the current moment by choosing a point on a fixed-size horizontal line where the ends are defined as the extremes (eg, *not at all* and *very much*). The VAS score is then determined by measuring the relative distance from the left end of the line to the participants’ chosen point.

##### External Evaluation With the TSST

Data from 122 participants who underwent the traditional TSST procedure were previously collected at Ruhr University Bochum in 4 independent studies [34,36-38]. The procedures included assessments of the affective responses using the Positive and Negative Affect Schedule (PANAS). We used the archived data and compared the effects of the TSST on the participants’ affective responses with the responses indicated by the DST participants in this study.

#### Statistical Analysis

Statistical analysis was performed using Python 3.7 (Python Software Foundation) with the pandas, statsmodels, and pingouin libraries. We assessed the distributions for normality and homogeneity of variances using Shapiro-Wilk and Levene tests, respectively. The participants’ affective responses were analyzed using mixed-model ANOVAs for repeated measurements with the factor *time* (baseline and after for the PANAS; baseline, intermediate, and after for the VAS) and the between-subject factor *group* (DST group vs C-DST group) separately for the PANAS and VAS scales. Owing to their robustness against deviations from the normality assumption [39], we also used ANOVAs for nonnormally distributed data. Greenhouse-Geisser corrections for df were applied where sphericity could not be assumed. Post hoc tests were performed using Bonferroni-adjusted Welch *t* test for different sample sizes and nonhomogeneity of variances [40].

To further analyze the DST parts regarding their stress induction potential, we calculated the mean VAS scores for every part of the DST or C-DST evaluated in the posttest questionnaire and descriptively ranked them.

To compare the affective responses of the participants performing the DST with those of the participants who underwent the TSST in previous studies, we analyzed the normalized scores for the PANAS positive and negative affect subscales using a 2-step meta-analysis. Therefore, we first performed paired *t* tests on the normalized pre- and post-PANAS scores for each of the TSST studies separately and calculated standardized effect sizes. Afterward, we computed a combined effect size for all TSST studies by assigning weights based on the inverse of the change score variance to the individual effect sizes of the respective studies [41] and compared it with the standardized effect size observed in DST participants.

In all analyses reported, we used 2-tailed comparisons with a *P* value of <.05 as the significance criterion. The effect size was reported using partial η^2^ for ANOVA and Cohen *d_z_* for paired *t* tests [42].

### Ethics Approval

Ethics approval for the study was granted by the University of Potsdam (application 33/2020), and the study was conducted in accordance with the General Data Protection Regulation. As this web-based study was conducted without experimenter supervision, special care was taken to ensure General Data Protection Regulation– and ethics-compliant informed consent, debriefing, and study cancellation process.

## Results

### Participants and Dropouts

Overall, 103 individuals completed the DST (50/103, 48.5% men; 52/103, 50.5% women; and 1/103, 1% other; mean age 31.34, SD 9.48 years), and 181 individuals completed the C-DST (83/181, 45.9% men, 96/181, 53% women, and 2/181, 1.1% other; mean age 31.51, SD 11.18 years). Most participants had a high level of education in both the DST (65/102, 63.7% had a university degree and 33/102, 32.4% had a high school degree) and C-DST groups (112/175, 64% had a university degree and 52/175, 29.7% had a high school degree). More details on the study participants’ ages and educational backgrounds can be found in Figures S1 and S2 in Multimedia Appendix 3.

The average time taken to complete the procedure was 7.69 (SD 1.35) minutes for the DST and 6.53 (SD 1.05) minutes for the C-DST. Most participants (263/284, 92.6%) did not report any usability issues.

Beyond the completed studies, 247 individuals started the study but dropped out. For the C-DST, 83.4% (206/247) of the initial participants completed the procedure, whereas 37.3% (112/300) completed the DST paradigm. The dropout rates at different time points during the procedure are shown in Figure 3. Most DST participants who did not finish the study had already dropped out before starting the Math-Task. Participants who did not complete the study were not included in the following analyses.

### DST Versus C-DST

**Figure 4:**
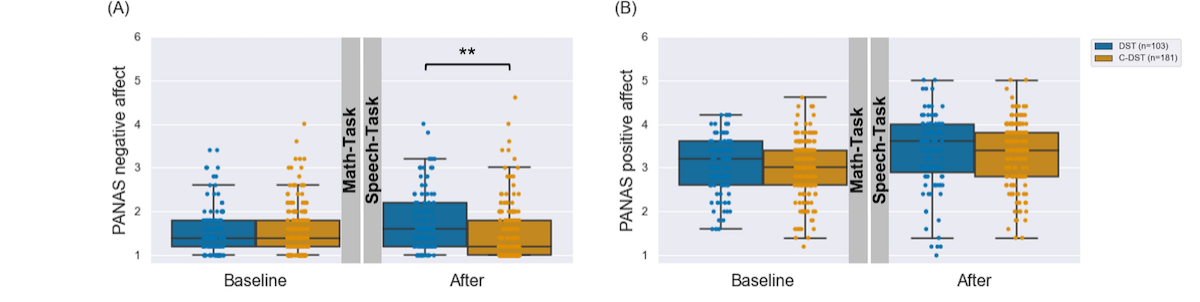
Negative (A) and positive (B) affect indicated in the Positive and Negative Affect Schedule (PANAS) subscales at baseline and posttest assessments for each participant in the Digital Stress Test (blue) and Control - Digital Stress Test (orange). A significant interaction between time and group was found for the negative but not the positive affect subscale. Digital Stress Test participants' negative affect was significantly higher at post-test assessment than Control - Digital Stress Test participants' negative affect (**P<.001 in post hoc Welch *t* test), whereas baseline scores did not significantly differ.

The DST and C-DST participants’ affective responses indicated in the PANAS questionnaires are shown in Figure 4. We found a significant main effect for the factor *group* (*F*_1,282_=5.83; *P*=.02; η*_p_*^2^=0.02) accompanied by a significant *group*×*time* interaction effect (*F*_1,282_=31.37; *P*<.001; η^2^=0.10) in the PANAS negative affect subscale. Post hoc analyses for the *group* effect showed that the participants’ overall reported negative affect was higher in the DST (mean 1.70, SD 0.55) than in the C-DST (mean 1.54, SD 0.57) group (*P*<.001). Post hoc tests for the *group*×*time* interaction effect revealed that the participants’ indicated negative affect did not significantly differ between the DST (mean 1.57, SD 0.56) and C-DST (mean 1.58, SD 0.58) groups in the baseline measurements (*P*=.99) but was significantly higher in the posttest assessments for DST (mean 1.84, SD 0.7) than for C-DST (mean 1.49, SD 0.64) participants (*P*<.001).

Conducting separate mixed-model ANOVAs for the participants’ indicated positive affect, we did not find significant *group* (*P*=.40) or *group*×*time* interaction (*P*=.51) effects, but we did find a significant *time* effect (*F*_1,282_=0.43; *P*<.001; η*_p_*^2^=0.002). The post hoc analysis showed that, overall, perceived positive affect increased in study participants (baseline: 3.02 –0.65 to +0.65; after the procedure: 3.34 –0.76 to +0.76; *P*<.001).

**Figure 5:**
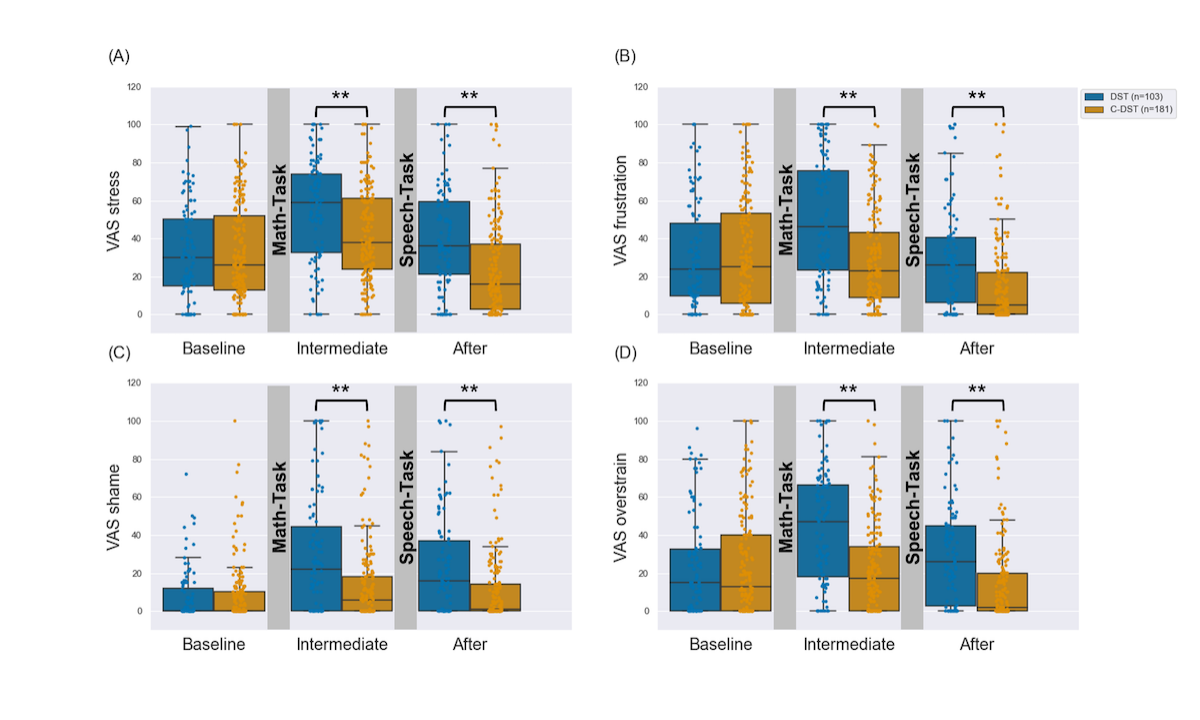
Visual Analogue Scale (VAS) responses for 4 different stress-related affect dimensions (A-D) of the Digital Stress Test (blue) and Control - Digital Stress Test (orange) groups at different times during the procedure. A significant interaction between time and group was found for all VAS scores. Subjective stress indexes were significantly elevated in the Digital Stress Test group compared with the Control Digital Stress test group at all time points after the baseline measurements (**P<.001 in post hoc Welch *t* test).

The participants’ responses to the 4 different VASs are shown in Figure 5. Regarding the *stress* scale, mixed-model ANOVAs revealed significant main effects for the factors *group* (*F*_1,282_=14.42; *P*<.001; η*_p_*^2^=0.05) and *time* (*F*_2,564_=75.11; *P*<.001; η*_p_*^2^=0.21) that were moderated by a significant *group*×*time* interaction effect (*F*_2,564_=14.28; *P*<.001; η*_p_*^2^=0.05). Post hoc analyses for the *group* effect revealed that overall reported stress responses were higher for the DST (mean 42.39, SD 20.87) than for the C-DST (mean 32.79, SD 20.27) participants (*P*<.001). Post hoc tests for the *time* effect showed that participants’ perceived stress significantly increased over the Math-Task (baseline: 32.92 –25.48 to +25.48; intermediate: 46.76 –26.01 to +26.01; *P*<.001) and decreased over the Speech-Task (intermediate: 46.76 –26.01 to +26.01; after the procedure: 29.15 –25.96 to +25.96; *P*<.001). Analyzing the *group*×*time* interaction, the participants’ indicated VAS scores were significantly higher in the DST group than in the C-DST group at all time points after the baseline measurements (*P*<.001 in all cases). Furthermore, we found very similar patterns for the 3 other stress-related attributes (*frustration*, *shame*, and *overstrain*) conducting separate mixed-model ANOVAs and post hoc tests (Multimedia Appendix 4).

### Analysis of Stress Elements

The results of the poststudy stress perception questionnaire are summarized in Table 1. According to the participants, the Math-Task was the most stressful element of the DST when compared with the framings in the beginning and with the Speech-Task.

Regarding the 2 tasks, the participants indicated the highest stress perception for the time pressure, whereas the social-evaluative component of being recorded through the front camera was not perceived as that stressful. Regarding the Math-Task, randomly swapping the input field after having correctly solved 3 calculation tasks seemed to induce a high level of perceived stress in the DST participants. Participants of the C-DST also rated the Math-Task and the implemented time limits as the most stressful elements of this version.

**Table 1:** Different parts of the Digital Stress Test (DST) and Control-Digital Stress Test (C-DST) and perceived stress levels sorted from highest to lowest indicating stress experience for each part of the DST paradigm.

Element in DST and C-DST and subcategory		Perceived stress—DST feedback (VAS^a^), mean (SD)	Perceived stress—C-DST feedback (VAS), mean (SD)
**Framing**			
	Participation in a performance test	65.4 (23.8)	46.7 (25.2)
	Behavior analysis through algorithm	40.6 (27.5)	N/A^b^
**Math-Task (overall)**		77.9 (18.4)	63.6 (23.7)
	Time limit	88.8 (13.5)	73.3 (21.7)
	Random input field swap	88.6 (15.6)	N/A
	Feedback after every calculation task	67.3 (24.0)	40.8 (27.8)
	Task difficulty	62.8 (26.3)	42.4 (23.0)
	Live comparison with other participants	59.8 (28.5)	N/A
	Personal performance	58.0 (29.0)	29.4 (22.3)
	Front camera activation	39.7 (28.6)	N/A
**Speech-Task (overall)**		46.2 (23.3)	25.5 (19.6)
	Preparation periods	45.5 (25.8)	19.6 (22.4)
	Time limits	45.1 (27.3)	22.6 (24.8)
	Questions	43.6 (23.8)	20.2 (22.7)
	Front camera activation	37.1 (25.9)	N/A
	Audio visualization of the voice	30.2 (24.2)	18.0 (21.8)

^a^VAS: Visual Analogue Scale.

^b^N/A: not applicable.

### DST Versus TSST

To further evaluate the stress induction potential of the DST, we performed a 2-step meta-analysis and compared the effects of the DST with findings of 4 previously conducted TSST studies. The results of each study are shown in Table 2. The sample sizes of the TSST studies ranged from 20 to 50, whereas 103 participants completed the DST in this study.

The participants’ indicated negative affect significantly increased in the DST and in all but one of the TSST studies (Table 2). The standardized effect sizes for the change in negative affect in the TSST studies ranged from 0.281 to 1.015, with a combined effect size of 0.667. The calculated effect for the increase in negative affect in the DST participants was 0.427.

The reported positive affect significantly increased in the DST participants in this study, whereas the results of the 4 TSST studies did not reveal significant changes in positive affect (Table 2). The standardized effect sizes for the change in positive affect in the TSST studies ranged from 0.022 to 0.363, with a combined effect size of 0.119. The calculated effect for the increase in positive affect in the DST participants was 0.382.

**Table 2:** Overview of studies used for meta-analytical comparison of the Digital Stress Test (DST) with the Trier Social Stress Test (TSST) effect including paired *t* test results for each study.

Study and PANAS^a^ subscale		Baseline score, mean (SD)	Score after, mean (SD)	Change, mean (SD)	Paired *t* test		
					*t* test (*df*)	*P* value	Cohen d_z_
**DST (n=103)**							
	NA^b^	1.57 (0.56)	1.84 (0.70)	0.27 (0.61)	−4.51 (102)	*<.001* ^c^	0.427
	PA^d^	3.08 (0.65)	3.37 (0.85)	0.29 (0.61)	−4.84 (102)	*<.001*	0.382
**TSST [37] (n=26)**							
	NA	1.28 (0.38)	1.82 (0.66)	0.55 (0.68)	−4.08 (25)	*<.001*	1.015
	PA	2.95 (0.45)	2.94 (0.59)	−0.01 (0.51)	0.11 (25)	.91	0.022
**TSST [34] (n=26)**							
	NA	1.33 (0.32)	1.66 (0.57)	0.32 (0.44)	−3.75 (25)	*<.001*	0.694
	PA	2.95 (0.56)	2.97 (0.64)	0.02 (0.45)	−0.17 (25)	.86	0.026
**TSST [36] (n=20)**							
	NA	1.36 (0.33)	1.51 (0.71)	0.16 (0.68)	−1.02 (19)	.32	0.281
	PA	2.75 (0.42)	3.02 (0.96)	0.27 (0.82)	−1.47 (19)	.16	0.363
**TSST [38] (n=50)**							
	NA	1.43 (0.56)	1.85 (0.72)	0.42 (0.53)	−5.64 (49)	*<.001*	0.655
	PA	3.02 (0.57)	2.88 (0.68)	−0.14 (0.47)	2.10 (49)	.04	0.221

^a^PANAS: Positive and Negative Affect Schedule.

^b^NA: negative affect.

^c^Italics emphasize significance.

^d^PA: positive affect.

## Discussion

### Principal Findings

In this proof-of-concept study, we evaluated the feasibility of a fully digitalized acute stress paradigm for smartphones, the DST, to induce and record psychosocial stress responses in outside-the-laboratory settings. We compared it with a digital control condition (C-DST) in a large web-based study and set the effect size of the participants’ indicated affect changes in the context of results previously achieved in the TSST. To our knowledge, this is the first study evaluating the stress reactivity of an experimenter-independent paradigm that does not include any human-human interaction.

We showed that the DST significantly induced higher levels of perceived stress and negative affect than the control condition. In addition to feeling more stressed, DST participants also reported similar increases in related affects such as frustration, shame, and overstrain. Notably, the reported increases in negative affect indicated by DST participants not only significantly exceeded those of participants performing the C-DST but were also comparable with those reported by TSST participants in previous studies regarding the calculated effect sizes.

These findings provide convincing evidence that an acute psychosocial stress response can be induced with a smartphone without any further equipment or experimenters taking part. In particular, the DST managed to induce subjective stress even if the social-evaluative threat and uncontrollability [25] of this study can be assumed to be weaker than in previous studies. TSST participants performed the paradigm in the laboratory and were administered physiological measurements and watched by several experimenters, whereas the DST and C-DST were mainly performed at home without any additional procedures or people present. Participants in the web-based study took <10 minutes for the whole paradigm and could cancel the study at any time by simply closing the browser. Nevertheless, the mere framing of social evaluation, a difficult mathematical task, and a free speech task in front of the smartphone camera were sufficient to elicit a psychological stress response.

These findings extend the results obtained in other studies analyzing the stress induction potential of less controlled and experimenter-dependent stress paradigms. Virtual reality versions of the TSST successfully elicit psychosocial stress responses using prerecorded [43], animated [44-47], or even nonhuman robot audiences [48]. However, these protocols still require experimenters to conduct the procedure.

Although previous studies have focused on the development of more immersive and convincing virtual realities to improve stress induction [49], our results indicate that the procedure might be simplified and spare human-human interaction. The recently investigated internet-delivered TSST has already shown that a significant stress response can be induced without direct person-to-person contact [16,17]. Our study supports these findings and further leads to the assumption that psychosocial stress can be induced without any live interaction.

Interestingly, in addition to evoking a significant level of perceived stress and negative affect, the DST also increased the participants’ positive affect. Increases in positive affect have also been reported in other studies, including stress tests [36,37]. We assume that the increase in this study was caused by an end-of-study relief and self-selection bias. First, the participants in this web-based study knew that the performance test would end after the last questionnaire, whereas, in many other studies, experimental measures or interventions followed the stress paradigm [14,50,51]. Second, participants with a strong decrease in their positive affect might have cancelled the study because of the very low cancellation barrier.

For a more detailed investigation of the stress induction potential of our new paradigm, we also examined the elements implemented for stress induction in the DST regarding the participants’ responses. Previous work has highlighted the impact of social evaluation and unpredictability on stress response. In particular, public speaking parts have been shown to induce stress in participants [25,52,53]. In our study, we found a strong increase in perceived stress throughout the Math-Task and a subsequent slight decrease over the Speech-Task. The results of the posttest questionnaires also indicate that the participants perceived the Math-Task as the more stressful task. In contrast to the TSST, the Speech-Task was the last part of the procedure, and the participants knew that the study would end afterward. Thus, the affect ratings might also have been influenced by the task order and end-of-study relief. Another reason for the lower stress induced by the Speech-Task might be that speaking to the front camera without any real social evaluation does not induce as much stress as that experienced in live experiments. Similarly, other paradigms that include a social-evaluative stressor without direct human interaction resulted in weaker stress responses [44,46,48]. In addition, despite receiving live feedback from the audio input, the participants might not talk or might skip the task as there is no real experimenter control. Furthermore, the participants in this study knew that their recordings would not be saved or watched.

For upcoming web-based studies, permanently saving the videos and the possibility that experimenters watch them might increase psychosocial stress. In addition, improving the credibility of the automated analysis through the implementation of more sophisticated adaptive feedback might lead to a stronger feeling of social evaluation. Another approach to strengthen the social-evaluative characteristics of the DST could be to implement a prerecorded or animated audience instead of displaying the participants’ own video recordings. In addition, strengthening the social comparison characteristics of the paradigm through fabricated comparisons of the performance during the Speech-Task (similar to the Math-Task) might lead to a stronger psychosocial stress induction.

### Web-Based Feasibility of the DST

The evaluation of the DST in a web-based study highlights the potential of this paradigm. Within 2 weeks, nearly 600 participants performed one of the versions, and almost 300 completed it. By contrast, a recent review evaluating 35 TSST studies showed that the average number of participants was 47, with only 1 study including >100 participants [54]. Campbell and Ehlert [55] evaluated 359 TSST and TSST-related articles and found only 6 studies that reported >100 participants, presuming many more laborious and time-consuming studies. Even in the recently proposed web-based TSST, experimenters and actors need to be present during the web-based videoconferencing session, and the still laborious procedure is stated as a limitation by the authors [16].

Another advantage of the DST procedure is its inclusiveness, allowing for participation from any location and in different conditions. However, the number and composition of the participants highly depend on the recruitment process. Many participants entered this study because of its announcement in a well-known German political podcast and university mailing lists, which might have led to age and educational background selection bias in our sample. The participants in this study were mainly younger and from higher educational backgrounds. Previous studies have shown differences in stress reactivity according to age and socioeconomic status, which need to be addressed when interpreting the findings of this study. In some studies, physiological stress responses to cognitive challenges were stronger in older and higher-educated individuals [56-58]. Nicolson et al [59] found stronger cortisol reactivity in younger individuals and no age-related differences in emotional responses to a speech task. According to Dickerson et al [25], cognitive testing may be more stressful for older adults with higher levels of education as they perceive a greater threat of negative social evaluation. Moreover, the average lower digital literacy of older adults [60] may even increase the stress response in older participants in a smartphone-based paradigm such as the DST. However, future studies should verify the stress induction potential for individuals of other ages and educational backgrounds.

In a web-based study without any direct supervision, it is crucial to ensure that the participants follow the correct procedure of the experiment. Therefore, the participants were automatically reminded to continue when they did not react during the tasks for a certain time. In addition, we logged the study progress and excluded participants who were extreme outliers with respect to the study duration. In the future, we plan to also analyze the video recordings regarding compliance and include more detailed live feedback.

The barrier for dropping out of this web-based study was much lower than that in laboratory or other live-contact settings. The participants could cancel the study at any time simply by closing the browser of their smartphones. Although it was ethically favorable that participants did not need to continue when they felt overwhelmed by the test situation, this also affected the outcome of the study. Many participants (324/547, 59.2%) dropped out even though the procedure took <10 minutes and no personal data were saved permanently. Most DST participants (89/300, 29.7%) had already cancelled during the introduction, which was not observed in the C-DST group. We assume that the higher cancellation rate in the beginning was caused by technological problems or privacy concerns related to the video recording in the DST.

For future versions, we plan to emphasize the high standard of data protection implemented in the DST and a cancellation procedure that allows for further decision-making regarding the submitted data and short feedback on the cancellation reasons.

### Limitations

Previous studies have highlighted the long-term consequences of acute stress–induced physiological changes [61], which were not evaluated in this study. Although, in some experiments, correlations between psychological and physiological stress responses could be found [62,63], others could not verify this [25,55,64]. Hellhammer and Schubert [65] found that psychological measurements during, but not before or after, the TSST were related to physiological responses. The DST participants reported the highest level of perceived stress between the 2 tasks, indicating that physiological changes might also have taken place. Even if it is not yet clear whether the stress response elicited by the DST entails physiological changes, addressing psychological stress reactivity plays an important role in the individual quality of life [66] and mental well-being [67]. Previous studies have shown the effects of interventions on psychological well-being [68,69], which highlights the potential use of the DST for evaluating stress intervention strategies.

Nevertheless, the stress induction potential of the DST should be confirmed in a follow-up study including measurements of other stress-relevant systems such as the sympathetic nervous system and the hypothalamic-pituitary-adrenal axis [55,70].

### Future Research

Several improvements to the stress induction procedure as well as the usability have been outlined. In particular, additional adaptive feedback algorithms that react to the participants’ live-recorded behavior might improve the credibility of the social-evaluative framing and enhance compliance.

To further validate the DST, we plan to compare the psychological and physiological stress responses, including cortisol, heart rate, and blood pressure measurements, of participants undergoing the TSST and DST in a within-subject design. Next, to improve and validate the stress induction procedure, we aim to adjust and evaluate the video data collection in the DST and build a large data set of stress test videos.

The DST might then be easily applied to different (clinical) cohorts (eg, stress in patients with chronic pain [71], stress in patients with cancer [72], and stress in students [73]) and contexts (eg, job stress [74] and parental stress [75]) from any internet-connected location worldwide. In contrast to existing protocols, this would also allow for the conduction of stress studies in outside-the-laboratory scenarios and with individuals from diverse cultural, ethnic, and geographical backgrounds (eg, remote cultures) [76].

In contrast, the multimodal video data collected using the DST could serve as the basis for the development of video-based stress analysis algorithms using machine learning methods [77]. Baird et al [78] combined 3 data sets including videos and voice recordings of participants undergoing the TSST in separate studies for the prediction of acute stress responses. Consequently, the data obtained with the DST could enrich existing video data sets and be used in combination with them (eg, pretraining for personalized models [79] and cross-model transfer learning [78]) to improve the quality of the algorithms. From a more long-term perspective, these algorithms might be used within the DST to provide feedback on a participant’s stress reactivity and evaluate personal prevention or intervention strategies (eg, resilience trainings [80]).

### Conclusions

To the best of our knowledge, this is the first approach to a standardized digital stress paradigm that can be carried out using only a smartphone. Moreover, our results imply that psychosocial stress can be induced through cognitive-verbal performance tasks and additional framings in a fully automated web application.

The ability to conduct (stress) studies without any experimenter or additional equipment required can also be seen as a potential turning point for translating traditional (stress) research to the wild. Owing to the web application–based mobile architecture, future researchers can quickly prepare, conduct, adapt, and evaluate studies anywhere—including basic and clinical research. In accordance with the principles of open access, the source code of the DST and C-DST is publicly available, and both applications can be freely used for research purposes upon request.

Future studies will evaluate the potential of the implemented video recording capability to provide a high-quality stress data set for algorithm development. This study may serve as inspiration to bridge the gap between classic psychological research and interdisciplinary computer science.

## Appendix

Multimedia Appendix 1 Video illustrating the procedure for the Digital Stress Test web application used in this study. The video has been modified to 2x playback speed, and recorded sound has been removed for publication purposes. For proper inspection or reading of specific parts, the video can be paused. Screen recordings were done by a research assistant in May 2021. The mock-up design was provided by Vectorium - de.freepik.com. A presentation version of the most recent digital stress test version without permanent data saving can be found at www.digitalstresstest.org.

Multimedia Appendix 2 Video illustrating the procedure for the Control - Digital Stress Test web application used in this study. The video has been modified to 2x playback speed, and recorded sound has been removed for publication purposes. For proper inspection or reading of specific parts, the video can be paused. Screen recordings were done by a research assistant in May 2021. The mock-up design was provided by Vectorium - de.freepik.com. A presentation version of the most recent control digital stress test version without permanent data saving can be found at www.digitalstresstest.org/control.

Multimedia Appendix 3 Distribution of age (Figure S1) and educational background (Figure S2) across Digital Stress Test and Control - Digital Stress Test participants.

Multimedia Appendix 4 Mixed-model ANOVA results for the comparison of participants’ affective responses indicated in the different VAS and PANAS questionnaires at different time points. Second sheet displays post-hoc analyses for significant effects.

## Abbreviations

API: application programming interface
C-DST: Control - Digital Stress Test
DST: Digital Stress Test
MIST: Montreal Imaging Stress Task
PANAS: Positive and Negative Affect Schedule
TSST: Trier Social Stress Test
VAS: Visual Analogue Scale
