# Supplementary figures and images for: Inducing and Recording Acute Stress Responses on a Large Scale With the Digital Stress Test (DST): Development and Evaluation Study
Source: J Med Internet Res. 2022 Jul 15;24(7):e32280. doi: 10.2196/32280 (PMC9338415; doi:10.2196/32280)

**Figure S1**

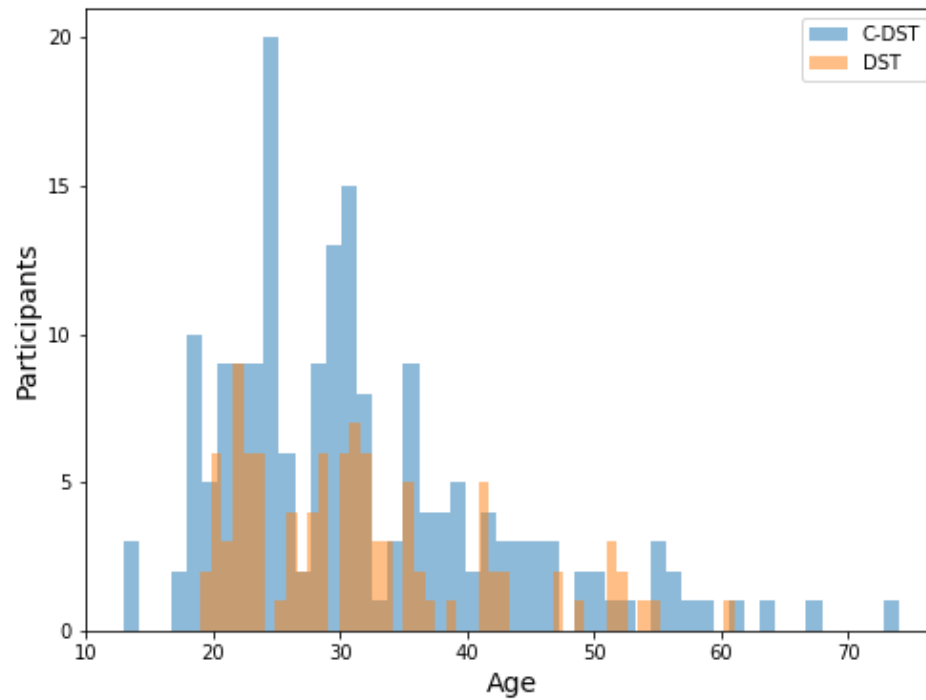

**Figure S2**

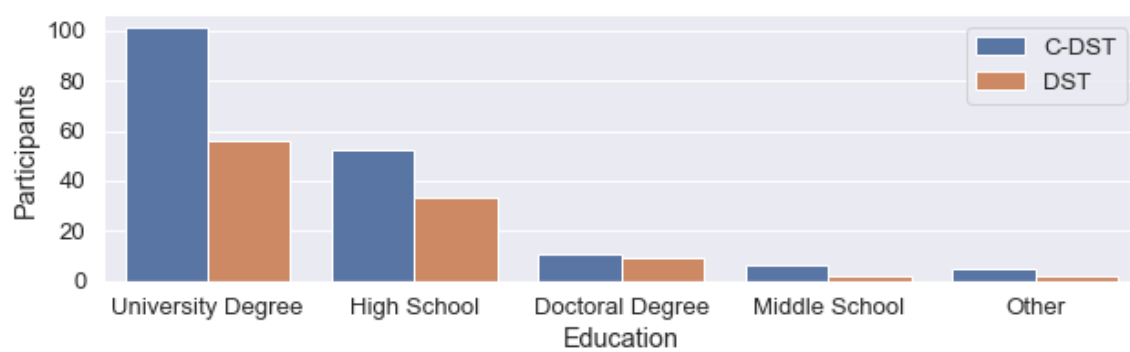

Supplement: Multimedia Appendix 3 [file jmir_v24i7e32280_app3.pdf]
